# High-Salt Diet Impairs the Neurons Plasticity and the Neurotransmitters-Related Biological Processes

**DOI:** 10.3390/nu13114123

**Published:** 2021-11-17

**Authors:** Xiaoyue Du, Lingqi Yu, Shengan Ling, Jiayu Xie, Wenfeng Chen

**Affiliations:** Institute of Life Sciences, College of Biological Science and Engineering, Fuzhou University, Fuzhou 350108, China; DXY2419326922@163.com (X.D.); yulingqi107@163.com (L.Y.); lsa2021121@163.com (S.L.); gtxiejy@163.com (J.X.)

**Keywords:** *Drosophila*, high salt diet, neurons plasticity, neurotransmitters

## Abstract

Salt, commonly known as sodium chloride, is an important ingredient that the body requires in relatively minute quantities. However, consuming too much salt can lead to high blood pressure, heart disease and even disruption of circadian rhythms. The biological process of the circadian rhythm was first studied in *Drosophila melanogaster* and is well understood. Their locomotor activity gradually increases before the light is switched on and off, a phenomenon called anticipation. In a previous study, we showed that a high-salt diet (HSD) impairs morning anticipation behavior in *Drosophila*. Here, we found that HSD did not significantly disrupt clock gene oscillation in the heads of flies, nor did it disrupt PERIOD protein oscillation in clock neurons or peripheral tissues. Remarkably, we found that HSD impairs neuronal plasticity in the axonal projections of circadian pacemaker neurons. Interestingly, we showed that increased excitability in PDF neurons mimics HSD, which causes morning anticipation impairment. Moreover, we found that HSD significantly disrupts neurotransmitter-related biological processes in the brain. Taken together, our data show that an HSD affects the multiple functions of neurons and impairs physiological behaviors.

## 1. Introduction

Sodium is an essential nutrient that contributes to many physiological processes, including the transmission of nerve impulses and normal cellular function [1]. However, excessive sodium consumption has been linked to health problems such as hypertension and heart disease [2]. Sodium is naturally present in various foods [3]. Most people consume excessive amounts of salt—on average, about twice the maximum recommended amount [4]. An HSD can also negatively impact brain health, causing deficits in behavior and physiological processes such as cognitive function [5,6,7] and sleep [8,9]. However, how an HSD affects brain neuron function is still unclear.

Many behavioral and physiological processes exhibit a 24-h rhythm that is regulated by the circadian rhythm system and can be altered by dietary components [10]. It has been reported that a diet high in fat and salt reduced locomotor activity and disrupted the diurnal rhythm of clock genes in the adrenal gland of mice [11]. An HSD shifts the phase of clock gene expression in peripheral tissues of mice [12]. A high-fat diet (HFD) has particularly detrimental effects on the circadian system [13]. The amplitudes of circadian clock gene expression were found to be decreased in the heart, liver, and kidney of Dahl salt-sensitive rats fed HSD compared to a group fed a normal salt diet [14]. Moreover, the circadian rhythm of plasma sodium could be disrupted in spontaneously hypertensive rats fed HSD [15]. This suggests that HSD disrupts the circadian clock and this could be a good way to study the effects of HSD on the neuron function.

The biological process of circadian rhythm was first studied in *Drosophila* and is best understood. Under light-dark (LD) conditions (usually 12 h of light: 12 h of dark), flies exhibit bimodal activity patterns that peak around the time the light is turned on (activity peak in the morning) and the light is turned off (activity peak in the evening) [16,17]. Their locomotor activity gradually increases before the light is turned on and off, a phenomenon called anticipation [16,17]. Transcriptional feedback loops are central to biological timing [16]. In *Drosophila*, the master clock consists in part of two interlocking feedback loops. In the main feedback loop, the transcription factor heterodimer CLOCK/CYCLE (CLK/CYC) binds to the promoters of the core clock genes period (*per*) and timeless (*tim*) to activate their transcription. In the second feedback loop, CLK/CYC directly activates the transcription of *vri* and *pdp1*. Another level of regulation is provided by the core clock gene clockwork orange (*cwo*). The five direct target genes of CLK/CYC (*per*, *tim*, *vri*, *pdp1*, and *cwo*), together with *clk* and *cyc*, are considered core clock genes and provide robust molecular circadian rhythms for the *Drosophila* molecular clock.

In the *Drosophila* brain, a network of about 150 clock neurons is organized for the maintenance of the circadian clock. The neuronal clock networks are divided into six groups based on their location and size. They are three groups of dorsal neurons (DN1s, DN2s and DN3s), three groups of lateral neurons (large LNvs (lLNv), small LNvs (sLNv) and LNds), and lateral posterior neurons (LPNs) [16,17]. The neuropeptide PIGMENT DISPERSING FACTOR (PDF), the important neurochemical of the circadian neuronal network, is expressed in the LNvs [18]. The sLNvs control the morning activity peak, while the LNds and the 5th sLNvs control the evening activity peak [19,20].

In a previous study, we found that flies given an HSD diet showed lower morning activity anticipations in normal light/dark in comparison to flies given a normal diet [8]. However, why an HSD impairs morning anticipation is still unclear. In this study, we hypothesize that an HSD disrupts the normal function of neurons in the brain. We found that an HSD impairs morning anticipation in *Drosophila* by regulating the plasticity of pacemaker neurons. In addition, we found that an HSD significantly inhibits the biological processes of neurotransmitter levels and their secretion or transport, and even inhibits the regulation of axonogenesis.

## 2. Materials and Methods

### 2.1. Fly Strains

The flies were kept at 25 °C unless otherwise stated. Line *w*^1118^ was used as a wild-type line [21]. The following lines were obtained from the Bloomington *Drosophila* Stock Center: UAS-mCD8::GFP (#5137), UAS-NaChBac (#9468), and UAS-TrpA1 (#26264). The *period*-EGFP fly was generated in our previous study [22]. The *pdf*-Gal4 line was provided by Dr. Yong Zhang (University of Nevada, Reno, NV, USA).

### 2.2. Locomotor Behavioural Assays

Three to five day old male flies were used for locomotor behavior analysis. Adult flies were fed 5% sucrose and 1% agar with or without 1% sodium chloride (Sigma-Aldrich, Catalog No. S1679) in monitor tubes [5(W) × 65(L) mm]. Light conditions were set at 12 h light and 12 h dark (12L:12D). Locomotor activity was monitored at 1 min intervals using a Trikinetics *Drosophila* Activity Monitoring (DAM) system (Trikinetics, Inc., Waltham, MA, USA) at the indicated temperature for at least 6 days. Activity data were analyzed using Microsoft Excel.

### 2.3. Quantitative Real-Time PCR

Three to five day old male flies were fed sucrose/agar food with or without 1% sodium chloride. After being treated for 4 days, 30 fly heads were collected at ZT1, 5, 9, 13, 17, 21 for RNA extraction. Total RNA was extracted using TRIGene (GenStar, Catalog No. P118-05) according to the manufacturer’s protocol. cDNA was prepared using HiScript III RT SuperMix (Vazyme, Catalog No. R323). qPCR was performed using RealStar Green Fast Mixture (GenStar, Catalog No. A301) on a LightCycler 96 (Roche). The following primers were used (*actin* was used as an internal control):

*per*-f: 5′-TGACCGAATCCCTGCTCAAT-3′

*per*-r: 5′-CTTTTTATCCCGTGGCCTGG-3′

*tim*-f: 5′-CACTTCCGCAACAACAGAGT-3′

*tim*-r: 5′-ACTCCGCAGGGTCAGTTTAA-3′

*clk*-f: 5′-GCAGGAAATCGCGTAATCTCA-3′

*clk*-r: 5′-ATCGGTGGCCTCATTATGATTTT-3′

*vri*-f: 5′-TATCGCCGACTCTCTCGATGA-3′

*vri*-r: 5′-CATTTGACTGCGGACTTATGGA-3′

*pdp1*-f: 5′-AAGGTAACACAATATGCCGACC-3′

*pdp1*-r: 5′-TGACCCAGGGAATCGGACC-3′

*cwo*-f: 5′-CTACGACGTTCACATGCAGGA-3′

*cwo*-r: 5′-GCTGGAGGCGCTTACATTATC-3′

*actin*-f: 5′-CAGAGCAAGCGTGGTATCCT-3′

*actin*-r: 5′-CTCATTGTAGAAGGTGTGGTGC-3′

### 2.4. Sodium Green Staining

The guts were dissected and incubated in a 5 μM cell permeable Sodium Green Tetraacetate Indicator (ThermoFisher, S-6091) at room temperature for 30–60 min. The guts were then transferred to 4% paraformaldehyde for 30 min. After a brief rinse with PBS for 5 min, the samples were mounted for image acquisition. The raw images were analyzed using ImageJ (NIH).

### 2.5. Immunohistochemistry

The brains of three-to five-day-old *pdf*-Gal4, UAS-mCD8::GFP male flies fed with or without 1% NaCl for 4 days were dissected at ZT2. The brains or malpighian tubules of three-to five-day-old period-EGFP males fed with or without 1% sodium chloride for 4 days were dissected at ZT1, 5, 9, 13, 17 and 21. Briefly, the whole flies were first fixed in 4% formaldehyde at 25 °C for 2 h. The flies were then dissected. Then the dissected brains or malpighian tubules were washed in PBT (PBS containing 0.2% Triton X-100) and blocked in 5% goat serum in PBT (PBST) for 30 min at room temperature. The primary antibodies mouse anti-PDF (DSHB, PDF C7) or rabbit anti-GFP (Thermofish, Waltham, MA, USA, # A-11122) were used to incubate the samples overnight at 4 °C. Finally, samples were washed three times with PBT and incubated overnight with Alexa Fluor 488-labeled goat anti-rabbit or Alexa Fluor 594-labeled goat anti-mouse secondary antibody (Thermofish) at 4 °C. Images of mounted samples were captured under confocal microscope (Leica TCS SP5) and analyzed using Image J (NIH).

### 2.6. Sample Collection and Preparation for RNA Sequencing

Three-to-five-day-old male *w*^1118^ flies were fed sucrose/agar diets with or without 1% NaCl for 4 days, and 50 individuals were subjected to head RNA isolation at ZT15. The sequencing libraries for transcriptome sequencing were made by Novogene (Beijing, China) and sequencing was performed using an Illumina Hiseq platform. Reads were mapped to the reference genome of *Drosophila melanogaster* dmel_r6.25_FB2018_06 released in Flybase (http://flybase.org/). The program featureCounts v1.5.0-p3 was used to count the number of reads mapped to each gene [23]. Then, the FPKM (expected number of fragments per kilobase of transcript sequence per millions base pairs sequenced) was calculated for each gene. Differential expression analysis of the control and 1% NaCl conditions (four biological replicates per condition) was performed using the DESeq2 R package (1.16.1) [24].

### 2.7. Gene Set Enrichment Analysis

Gene Set Enrichment Analysis (GSEA) was used to determine the coordinated expression within treated samples of a priori defined groups of genes [25,26]. The GSEA analysis processes were performed with R v4.10. Basically, all differential expression genes between control and 1%NaCl conditions were used as input. The log2FoldChange, which indicates how much the transcript’s expression changed between the 1%NaCl and control groups, was used to create a list of genes with gene names and values in descending order. The gene sets of *Drosophila melanogaster* were obtained from the Molecular Signatures Database (MSigDB) using the msigdbr package. Then, the clusterProfiler package, a universal enrichment tool for interpreting omics data, was used to perform the enrichment analysis [27].

### 2.8. Statistical Analysis

The Mann–Whitney U test was used to compare two columns of the 1%NaCl and control groups’ data with Prism 7 (GraphPad Software).

## 3. Results

### 3.1. Sodium Levels Increased in the Flies with HSD

**Figure 1:**
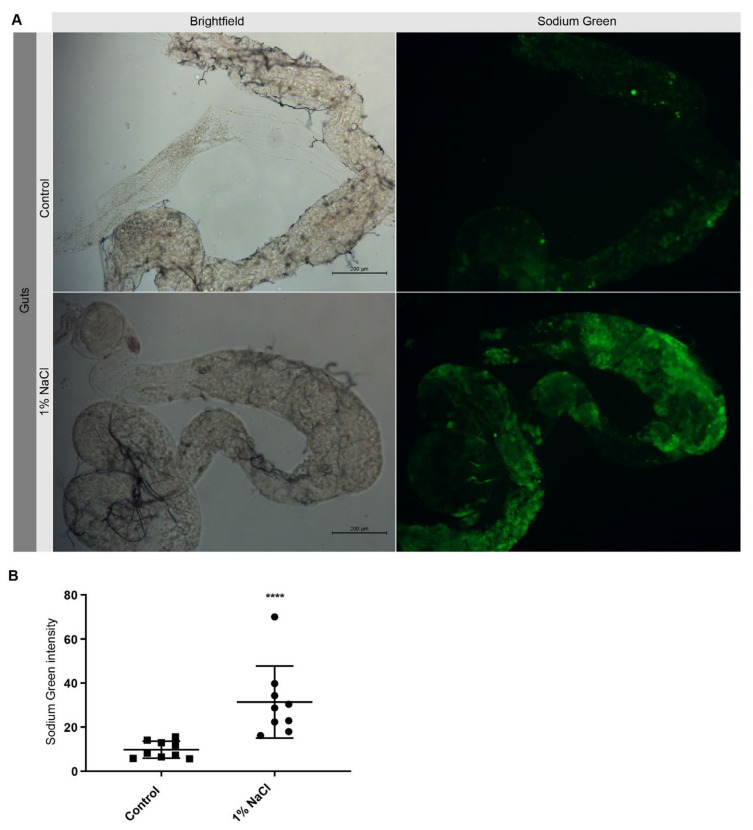
Elevated Na^+^ in the midgut of HSD flies. (**A**) Na^+^ concentration in midgut of control and HSD flies indicated by Sodium Green staining. (**B**) Quantitative measurements of Sodium Green intensity. **** *p* < 0.0001.

To make sure that an HSD did indeed result in an increase in Na^+^ in the flies, we used Sodium Green to monitor Na^+^ in the midgut. Sodium Green is a Na^+^-sensitive probe for fluorometric determination of Na^+^ concentrations with sufficient selectivity [28]. Here, male flies were fed diets containing 1% NaCl or normal diet under 12L:12D cycles for 4 days and then the midguts were dissected at ZT2 for Sodium Green staining. In a previous study, we performed an ion chromatography assay to determine the chloride ions concentration in the flies and found that the chloride ions were nearly four times higher in flies fed HSD than in the control group [8]. Here, using the Sodium Green assay, we found that HSD flies had a higher concentration of intracellular Na^+^ in the midgut, as shown by the increased fluorescence (Figure 1A,B). Overall, this suggests that HSD flies are indeed ingesting the high-salt diet.

### 3.2. HSD Slightly Decrease the Clock Genes Oscillation Amplitude in the Fly Heads

**Figure 2:**
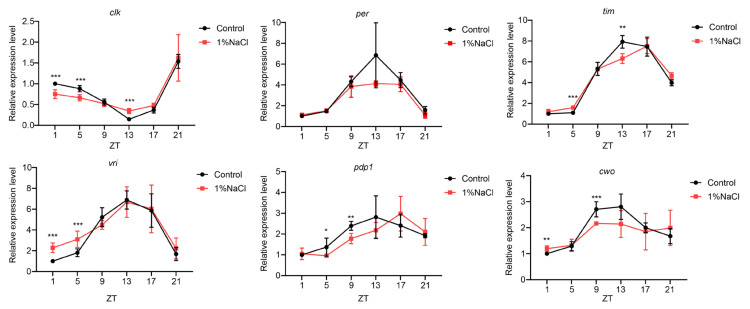
Oscillation of clock gene expression in head extracts of flies with or without HSD. An average of the normalized values was determined and plotted. RNA profiles were from 12L:12D cycles. The Mann-Whitney test was used to compare data at the same time point between control and flies fed 1% NaCl. * *p* < 0.05; ** *p* < 0.01; *** *p* < 0.001.

Morning and evening activities are controlled by the circadian clock, as inferred from the loss of morning and evening anticipatory activity in *per*^0^ mutants [29]. To test whether an HSD affects the expression of core clock genes and contributes to the impairment of morning anticipation, we examined the expression of the clock genes *clk*, *per*, *tim*, *vri*, *pdp1* and *cwo* in an HSD and in control fly heads with samples taken at ZT 1, 5, 9, 13, 17. While flies fed with an HSD still show oscillation of clock genes in their heads, we found that HSD flies tend to slightly decrease the amplitude of oscillation of clock genes compared to controls (Figure 2). This suggests that HSD may impair the flies’ morning anticipation by disrupting the robustness of the oscillation of the core clock genes in the flies’ heads.

### 3.3. HSD Neither Alters PERIOD Protein Oscillation in the Clock Neurons Nor in the Peripheral Tissues

**Figure 3:**
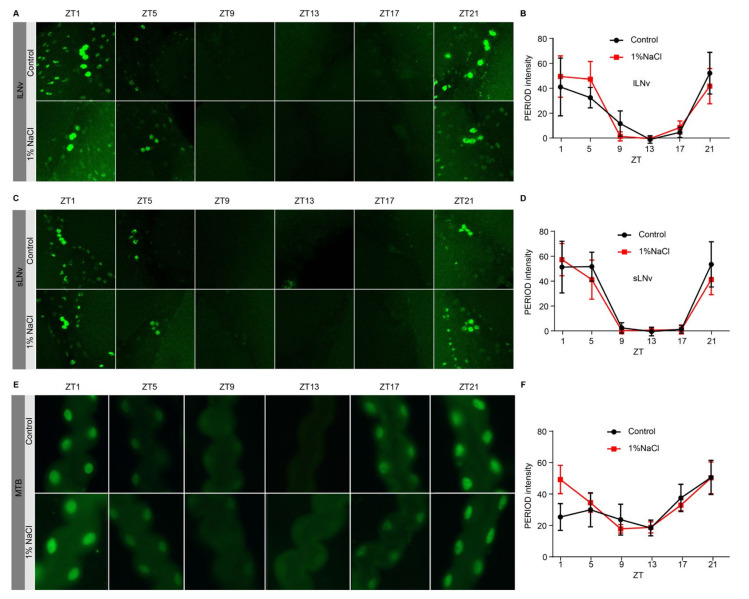
Oscillation of PER in period-EGFP flies with HSD and normal diet. (**A**,**B**) Oscillation of PER in the lLNvs. (**C**,**D**) Oscillation of PER in the sLNvs. (**E**,**F**). Oscillation of PER in the Malpighian tubules.

To further investigate the effects of HSD on rhythmic clock protein expression, we also examined the 24-h expression profiles of PERIOD proteins in brain clock neurons. To study PERIOD protein oscillation, we used a period-AID-EGFP fly whose EGFP tag was fused to the endogenous period locus after the region encoding the C-terminus of the protein [22]. Period-AID-EGFP flies were fed a normal diet or an HSD for 4 days and dissected at specific time points for GFP staining on the fifth day. Previous genetic dissections of clock neurons have shown that the sLNvs are responsible for morning activity [19,20], so we decided to observe PERIOD protein oscillation in the LNvs. However, we found no significant difference in the circadian oscillatory expression of the PERIOD protein in the sLNv or lLNv between the HSD and control flies (Figure 3A–D). Since it has been reported that an HSD alters molecular circadian rhythms in peripheral tissues of mice [10,12], we wanted to determine whether an HSD affects the expression rhythm of PERIOD protein in peripheral tissues of *Drosophila*. Since Malpighian tubules are one of the peripheral tissues operated by the circadian clock [30], we monitored the levels of PERIOD in Malpighian tubules and observed similar oscillations of PER signals in both HSD and control (Figure 3E,F). Both results suggest that an HSD may regulate the morning activity by modulating the output pathway.

### 3.4. An HSD Impairs the Neurons Plasticity in the Axonal Projections of Circadian Pacemaker Neurons

**Figure 4:**
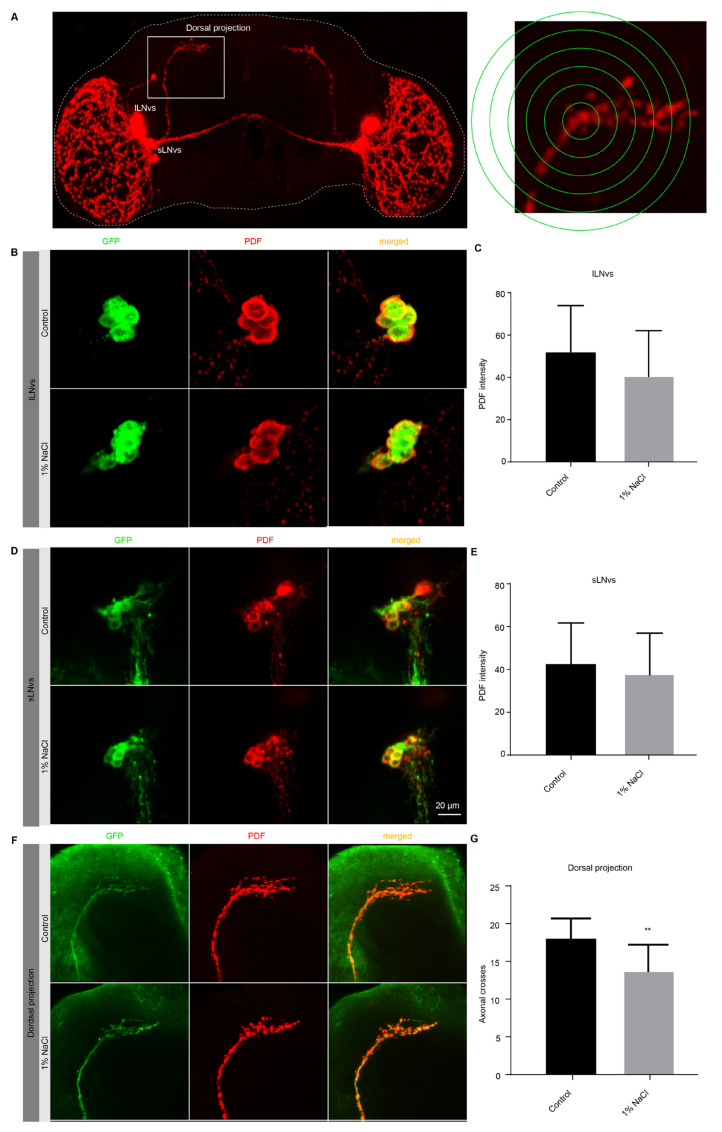
Daily reorganization in PDF terminals is reduced by an HSD. (**A**) Brains were stained with anti-PDF antibodies to show sLNvs, lLNvs, and quantification of axonal PDF projection terminals. (**B**–**E**) Staining and quantification of PDF content in sLNvs and lLNvs. pdf>mCD8-GFP wild-type brains were fed HSD or normal diet and dissected at ZT2. (**F**,**G**) Staining and quantification of axonal projection terminals of PDF in flies fed HSD or normal diet. ** *p* < 0.01.

The neuropeptide PDF is essential in LNvs for the control of rest-activity cycles in *Drosophila*. It has been previously shown that sLNv neurons exhibit diurnal remodeling of their dorsal axonal projections under clock control [31], a phenomenon termed circadian structural plasticity (Figure 4A). To test whether HSD affects the circadian plasticity of PDF-positive sLNv neurons, we examined the terminal of dorsal sLNv projections in HSD and control flies using a PDF-specific antibody at ZT2. Based on the whole mount PDF immunohistochemistry in flies brains (Figure 4B–E), we did not detect a difference in PDF content between HSD and control flies in either sLNvs or lLNvs. Remarkably, we found that the flies fed with normal diet had more branches of axon terminals at ZT2 than the flies fed with HSD (Figure 4F–G). These results suggest that HSD impairs the remodeling of sLNv axon terminals.

### 3.5. Enhancement of Excitability in PDF Neurons Mimics the HSD That Causes Morning Anticipation Impairment

**Figure 5:**
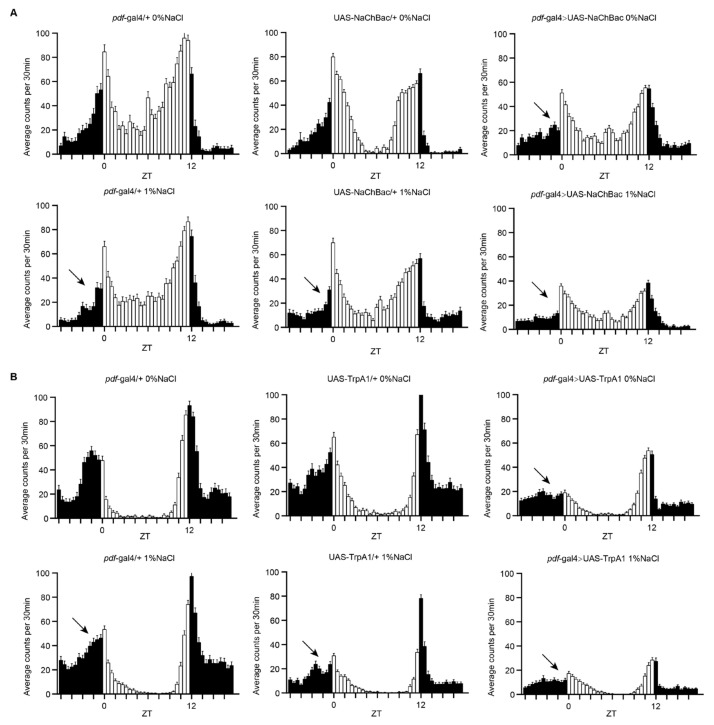
Enhancement of neuronal function in PDF-expressing neurons mimics the HSD that causes impaired morning anticipation in flies. (**A**) Locomotor activity of the indicated strains measured during three days under LD conditions. *pdf*-Gal4 drives UAS-NaChBac expression and controls (*pdf*-Gal4/+ and UAS-NaChBac/+) were fed HSD or normal diet. (**B**) Locomotor activity of the indicated strains measured during three days under LD and 29 °C conditions. *pdf*-Gal4 drives UAS-TrpA1 expression and controls (*pdf*-Gal4/+ and UAS-TrpA1/+) were fed HSD or normal diet. Arrows indicate perturbed morning anticipation.

The increase in action potential in excitable tissues is the result of a large and rapid Na^+^ influx [32]. It has been reported that high salt intake increases the excitability of some neurons in rodents [33,34]. We therefore assumed that HSD might disrupt morning anticipation behavior by increasing excitability in PDF neurons, and the GAL4-UAS system is the appropriate tool to test this assumption [35]. To selectively increase the excitability of PDF neurons, we used transgenic flies with *pdf*-Gal4 driver and flies containing a UAS-transgene for the voltage-activated bacterial sodium channel NaChBac, which is a novel tool to selectively enhance neuronal excitability [36]. Similar to control flies fed an HSD, morning anticipation was abolished in *pdf*-Gal4-driven NaChBac expression flies (Figure 5A). To rule out a developmental effect, we also used the temperature gated TRPA1 cation channel to activate PDF neuronal circuits in the adult stage [37]. Interestingly, the sustained activation of PDF neurons triggered by the expression of UAS-TrpA1 at the restrictive temperature also elicited an impairment of morning anticipation that mimicked control flies fed an HSD (Figure 5B). These results suggest that an HSD may impair morning anticipation by increasing the excitability of PDF neurons.

### 3.6. An HSD Disrupts the Neurotransmitters-Related Biological Processes in the Brain

**Figure 6:**
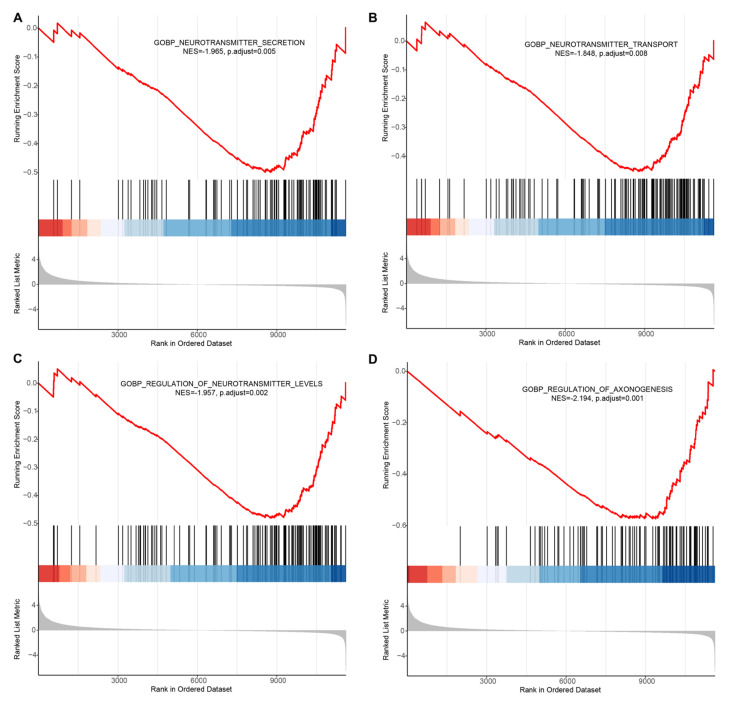
GSEA enrichment analysis. (**A**) Biological process of neurotransmitter secretion. (**B**) Biological process of neurotransmitter transport. (**C**) Biological process of neurotransmitter levels regulation. (**D**) Biological process of axonogenesis regulation. In GSEA enrichment graphs, the curves represent the running sum of enrichment scores, the middle part of the graph shows the position of genes associated with specific pathways, and the bottom part of the graph shows how the fold change is distributed along with the gene list. The normalized enrichment score (NES) and the adjusted *p*-values were shown in the graphs.

To gain further biological insight into HSD in the brain, we performed RNA-seq analysis to identify key functional gene sets that correlate with HSD. Functional enrichment analysis is one of the most commonly used techniques to interpret gene lists derived from various high-throughput studies [27]. Here, we performed GSEA for the control and 1%NaCl data (see Table S1, Supplementary Material) using the functional gene sets of *Drosophila melanogaster* from MSigDB [25,26]. For the NaCl > control comparison, we identified 145 sets whose expression was correlated with an HSD (see Table S2, Supplementary Material). Interestingly, we found that an HSD significantly inhibited neurotransmitter levels and their secretion or transport (Figure 6A–C). In addition, a set of axonogenesis regulation, which is any process that modulates the frequency, the rate or the extent of axonogenesis, as well as the generation of an axon and the long process of a neuron, was also inhibited by an HSD. Taken together, the results show that an HSD alters the state of gene expression in the brain at a global level, and severely affects the normal function of neurons, especially biological processes related to neurotransmitters.

## 4. Discussion

HSD has been shown to reduce the amplitudes of circadian expression changes in clock genes in the heart, liver, and kidney of Dahl salt-sensitive rats fed a high-salt diet, which is consistent with our findings [14]. It is also known that peripheral clocks reside in various organs and tissues, therefore we cannot rule out that a HSD may affect the biological clocks of other peripheral tissues [38].

High salt intake may be linked to the declining of brain function. Previous research revealed that an HSD inhibited the production of nitric oxide (NO), which helps direct blood vessels to relax [7]. As a result of the lower NO from the HSD, mice have poorer cognition and restricted blood flow in the brain [7]. However, the effect of reduced blood flow was not enough to directly cause the loss in cognitive abilities [39]. High quantities of dietary salt were reported to produce an accumulation of phosphorylated tau protein in the brain [39]. Furthermore, an HSD can raise levels of the glucocorticoid hormone corticosterone, which helps to control the permeability of the blood–brain barrier and the entry of inflammatory T cells into the central nervous system (CNS) [40]. Intriguingly, a high-salt maternal diet lowers cerebellar mitochondrial mass and membrane potential, changing the redox state of the offspring’s brain [41].

The natural function of neurons is also harmed by excessive salt consumption. High dietary salt intake, for example, enhances the expression and function of epithelial Na^+^ channels (ENaCs), resulting in vasopressin (VP) neurons depolarizing at steady state [42]. Previous work has shown that an HSD impairs the dendritic spine structure or synaptic plasticity. In comparison to wild type control rats, high saline-administered rats showed a significant reduction of dendritic spine density in the hippocampus [6]. In addition, an HSD also reduces memory-related synaptic plasticity by increasing oxidative stress and decreasing synaptic protein expression [43]. In animals subjected to an HSD, analysis of quantal excitatory synaptic events indicated pre- and post-synaptic alterations that would enhance MNCVP excitation [34]. Here, our data provide the evidence that an HSD could impair the *Drosophila* brain circadian pacemaker neurons plasticity, and HSD impairs the neurotransmitters release or transport. Neurotransmitters are also referred to as the body’s chemical messengers. They are the signaling molecules used by the nervous system to transmit messages between neurons, or from neurons to other cells across a synapse. How many other behavioral and physiological processes are affected by an HSD remains to be further studied.

Too much salt may speed up the aging process. A decline in circadian output behavior and sleep is linked to age-related alterations in clock neuron structural plasticity and excitability. In wild-type flies, morning and evening anticipation decreases with age, and sLNv terminal remodeling decreases with age [44]. This is consistent with our findings that HSD flies show an inhibition of morning anticipation behavior and sLNv terminal remodeling. Moreover, in our previous study, an HSD has been found to cause sleep fragmentation as they shown in aging flies [8,45]. High sodium intake speeds up the cellular aging process, which is associated with short leukocyte telomere length [46]. An HSD may impair the neurons functions in the brain by the aging process.

## Data Availability

All data is available in this paper.

## Supplementary Materials

The following are available online at https://www.mdpi.com/article/10.3390/nu13114123/s1, Table S1: Differential expression genes analysis of control and 1%NaCl conditions; Table S2: Gene sets enriched by the GSEA among 1%NaCl > control conditions.
